# Changing the culture: a qualitative study exploring research capacity in local government

**DOI:** 10.1186/s12889-022-13758-w

**Published:** 2022-07-14

**Authors:** Catherine Homer, James Woodall, Charlotte Freeman, Jane South, Jo Cooke, Judith Holliday, Anna Hartley, Shane Mullen

**Affiliations:** 1 Sport and Physical Activity Research Centre, Sheffield Hallam University, Olympic Legacy Park, 2 Old Hall Road, Sheffield, S9 3TU UK; 2 School of Health, Leeds Beckett University, Portland Building, Leeds, LS1 3HE UK; 3 Health Sciences School, Division of Nursing and Midwifery, University of Sheffield, Barber House Annex, 3a Clarkehouse Road, Sheffield, S10 UK; 4 Research Department, Mid Yorkshire Hospitals NHS Trust, Pinderfields Hospital, Aberford Road, Wakefield, WF1 4DG UK; 5 Wakefield Council, Wakefield One, Burton Street, Wakefield, WF1 2EB UK

**Keywords:** Local government, Research capacity, Embedded researcher, Research system, Public health, Qualitative, Evidence based practice

## Abstract

**Background:**

Local government has become a key constituent for addressing health inequalities and influencing the health of individuals and communities in England. Lauded as an effective approach to tackle the multiple determinants of health, there are concerns that generating and utilising research evidence to inform decision-making and action is a challenge. This research was conducted in a local authority situated in the north of England and addressed the research question – ‘What is the capacity to collaborate and deliver research?’. The study explored the assets that exist to foster a stronger research culture, identified barriers and opportunities for developing research capacity, and how a sustainable research system could be developed to impact on local residents’ health and reduce health inequalities.

**Methods:**

This was a qualitative study utilising semi-structured interviews and focus groups. The study used an embedded researcher (ER) who was digitally embedded within the local authority for four months to conduct the data collection. Senior Managers were purposively sampled from across the local authority to take part in interviews. Three focus groups included representation from across the local authority. Framework analysis was conducted to develop the themes which were informed by the Research Capacity Development framework.

**Results:**

Tensions between research led decision making and the political and cultural context of local government were identified as a barrier to developing research which addressed health inequalities. Research was not prioritised through an organisational strategy and was led sporadically by research active employees. A recognition across leaders that a culture shift to an organisation which used research evidence to develop policy and commission services was needed. Building relationships and infrastructure across local government, place-based collaborators and academic institutions was required. The embedded researcher approach is one method of developing these relationships. The study identifies the strengths and assets that are embedded in the organisational make-up and the potential areas for development.

**Conclusion:**

Research leadership is required in local government to create a culture of evidence-based principles and policy. The embedded research model has high utility in gaining depth of information and recognising contextual and local factors which would support research capacity development.

## Background

The role that local government can play in improving population health is recognised Internationally. Yet, there are very limited research systems that exist within local government to support their ability to create and synthesis the evidence needed for preventative and public health interventions. Most research systems exist outside of the local authority and are based within health, community and academic partnerships [1]. In England, local government has become a key constituent for addressing health inequalities and influencing the health of individuals and communities [2]. While this has been lauded as an effective approach to tackle the multiple determinants of health, there are concerns that generating and utilising research evidence to inform decision-making and action is a challenge [3, 4]. This situation is not isolated to England and international reviews have shown various ways in which local government access and acquire evidence for decision-making – one review suggesting six models and approaches between local government and research systems [1]. Indeed, evidence-informed decision-making is complicated and involves integrating the best available research evidence with contextual factors including community preferences, local issues, political preferences and public health resources [5]. With this backdrop, this paper reports research which sought to understand the capacity to collaborate, deliver and utilise research across one metropolitan district council. The research explored current assets within local government in relation to research development and evidence implementation and how these could be further harnessed. Moreover, the research identified limitations and shortcomings which prevented research use and activity from flourishing. The paper draws out implications more widely for local government and how to reconfigure the relationship between research, evidence and decision-making in public health.

The transfer of public health functions in England from the National Health Service (NHS) to local government in 2013 aimed to bring about improvements to population level health and to reduce health inequalities. While the delivery of public health can vary in local authorities [6], this reorganisation saw a change in culture from a narrow focus on health care pathways to one of a politically led environment with opportunity to influence the wider determinants of health and wellbeing. As part of this, Health and Wellbeing strategies are a vehicle for local governments to act on the wider determinants of health and wellbeing and provide an opportunity to adopt an evidence-based approach to local decision making and prioritisation of limited resources across local government. Nonetheless, the use of evidence and published research within these strategies is not common practice. Analysis of Health and Wellbeing strategies by Beenstock et al. [7] identified that only five out of 47 Health and Wellbeing strategies referred to published research evidence and only three cited National Institute for Clinical Excellence (NICE) guidance.

Barriers to the use of research and evidence to guide decision making include the questioning of the credibility of the evidence [3, 4] and the transferability of evidence that is out of context and not generated in the local setting [3]. Studies exploring how evidence in local authority public health practice is used have highlighted the disconnect in understanding between policy makers and academics, especially in regard to what constitutes robust and useful knowledge [8]. Indeed, locally generated data are viewed by decision makers as fitting the political context, having more transferability, and thus having a bigger influence on their local decision making [9]. In addition, a systematic scoping review exploring the use of evidence in local public health decision making concluded that researchers need to develop a deeper understanding of evidence requirements from the perspective of decision-makers [10].

The Local Government Association (LGA) [11] further reiterated the value of research in local government settings. The LGA recently highlighted that ‘local government needs practical research providing solutions that can be applied in real world situations. Councils can benefit from engaging in research partnerships’ (p.8). The report suggests the need for increased capacity and development of the local authority research system. Other reports have also signalled the importance of taking a population level, non-clinical and transdisciplinary approach to public health interventions and research [12]. How that vision translates into practice and work ‘on the ground’ is relatively under-explored and understood. So, while the rhetoric is strong, it is clear that there are significant challenges based on the “daily rush to support frontline delivery of services with a lack of resources” (p.8) in local government. This means that time, expertise, and space to use or generate research is a struggle [13].

In the UK the National Institute of Health Research (NIHR) funds health and social care research that aims to improve people’s health and wellbeing. The NIHR recognised the position that local government can have to improve population health and set out a funding call (in April 2020) to identify how local authorities could be developed into locally based research systems and to shape future investment. The research presented here was conducted following a successful application to the local authority research system funding call. The research was based in one local authority in the north of England where qualitative methodology was employed, operationalised through interviews, focus groups, meeting observations and documentary review. This paper focuses specifically on interviews and focus groups with a range of local authority personnel (described in more detail shortly) to enable greater understanding of the capacity of the local authority to collaborate and deliver research.

## Methods

The research was undertaken between August–November 2020. The overarching aim was to explore the current research assets in the local authority and to determine how these could be nurtured and replicated within the organisation to foster a stronger research culture. In addition, the research sought to identify any perceived barriers that exist to the local authority working with academic partners. In particular, to establish research capacity and opportunities, and explore with key members of the organisation how a sustainable research system could be developed to impact on local resident’s health, reduce health inequalities and identify the most important research outcomes. The theoretical underpinning of the research was the Research Capacity Development Framework [14].

The study adopted a collaborative approach throughout from the funding bid development to outputs and dissemination. A project steering group was established which included representation from: the local authority at strategic, operational and political levels, neighbouring local authorities who had also received NIHR funding, local academic intuitions, NHS research infrastructure support networks and the local NHS hospital trust. This steering group supported with the study design, recruitment and data analysis and, following the study, knowledge transfer and dissemination. The study was chaired by an elected member. In the UK an elected member is chosen to represent their local area and inform and influence the decisions and running of the local authority. Elected members may have key responsibility for different portfolios such as health, children’s services, planning and transport.

Data collection was undertaken by an Embedded Researcher (ER) who was based within the local authority for the study period. The ER model is becoming increasingly highlighted as allowing a joined-up approach to creating and using knowledge by placing a researcher in a non-academic organisation to better link research and practice [15]. The decision to use an ER in this study was so that it could potentially provide greater depth and insight within an organisation through having a researcher integrated within the culture and environment. However, this was compromised during the Covid-19 pandemic and the ER became digitally, rather than physically, embedded in the local authority. As part of the ER process, a co-applicant of the study facilitated access for the ER to attend to attend online team meetings at the operational and strategic level with various departments across the local authority in order to meet employees, develop a rapport with teams and raise awareness of the study. This included attending team meetings and formal committees. The ER was introduced to strategic directors by another co-applicant of the study who was also a member of the Local Authority leadership team. Prior to the study starting the strategic leadership were informed and supportive of the study, this helped with rapport building in preparation of the interviews. While the research team conceded that the original intention was for the ER to be co-located in situ with staff in the local authority, there was still methodological learning and value from a digitally ER working within the organisation. This is reflected upon later.

### Setting

The research focused on a single local authority in the north of England. The area is one of the largest Metropolitan Districts in the country and is one of the largest cities in the UK, without its own university, with levels of educational attainment below average. The area is in the top twenty-percent of the most deprived districts in England and on average, people die younger than in other parts of England. Cardiovascular, cancer and respiratory illnesses are in high levels in the district resulting in people becoming ill at a younger age, having to live with their illnesses longer compared to most of the rest of the country.

### Sample

Purposive sampling was used for both identifying individuals for the interviews and focus groups. The sampling was conducted with support of the project steering group in which a discussion was had to identify the key strategic roles and groups from across the authority that would need to be included. The steering group also identified groups of people who were research active (involved in delivering or commissioning research or who held a research related qualification), roles within public health where research was considered to be used in practice on a regular basis, and elected members who had responsibilities for different portfolios across the local authority. Participants were recruited via email invitation. All participants were provided with a briefing paper, written by members of the project team and co-applicants employed by the local authority, and a participant information sheet, prior to data collection to ensure informed consent was gained. Consultation with the study steering group informed the sampling of three focus groups which were conducted with: Focus Group 1 - Elected Members (*n* = 3), Focus Group 2 - Public Health Officers (*n* = 6) and Focus Group 3 - Officers with research interests across the local authority (*n* = 4). Interviews (*n* = 7) were conducted by the ER with Corporate Directors and Service Managers purposively sampled to enable the research questions to be explored fully.

All data collection was undertaken online using Microsoft Teams due to social distancing restrictions of the Covid-19 pandemic. All aspects of the study received ethical approval from both Leeds Beckett University and Sheffield Hallam University and access permissions were gathered from the local authority via the strategic leadership team. Interviews and focus groups were conducted in parallel due to the short time frame in which to conduct the research and lasted between 30 and 60 minutes. Interviews and focus groups explored a range of issues which were informed through the Research Capacity Development (RCD) framework developed by Cooke [14, 16]. The RCD works at individual, organisational and systems levels, with a purpose to develop research that is useful and impactful to society [17, 18]. Assessing both the assets and potential for RCD of an organisation can help articulate what a partner may bring to a collaboration and can be considered an important aspect of win-win research partnerships. The RCD framework has been applied in a range of contexts and in developing of organisational research strategy [16].

Using the principles of the RCD framework [18] the interview schedules and focus group guides covered: linkages and partnership; skills and confidence in the workforce and wider community; infrastructure of the council and wider partnerships, research use and dissemination, experience and assets of coproduction in projects (including citizen and public engagement in projects); and ownership, leadership and sustainability of research activity (both by Officers and Elected Members).

### Data analysis

Interview and focus group recordings were transcribed by an external transcription company, anonymised and shared as a secure online file which was accessible by three members of the research team. All transcripts were coded on NVivo 12 by the ER and two members of the research team cross checked a sample for coding accuracy. Data were analysed using framework analysis [19]. Framework analysis was used as an expedite method given the short timescale for the project funding and was deductively informed following the RCD framework [14]. Specific elements of the RCD framework were used in the development of the matrices – a core aspect of framework approach – this seemed pragmatic in deductively analysing the data set given the RCD framework was used to inform the data collection tools (as discussed earlier). Given the limited timeframe set by the funder for the research delivery, the data was analysed sequentially with interview analysis being completed first followed by focus groups. This was based on pragmatics, but also was beneficial in refining analytical categories and themes during the process and supported the triangulation of the two sets of data. Inductive coding and inductive thematic development was also part of the analytical process to enable specific ‘local’ issues within the local authority to be represented.

## Results

The analysis revealed a range of thematic areas relating to the focus of the research. This section presents these to highlight the barriers and potential in local authorities for improved, research-led, decision making to address health inequalities.

### Barriers

Respondents identified challenges to improving research-led decision making to address health inequalities.

#### The political and cultural context

Respondents described a duality in the use of research and evidence within local authority decision making, and how the essential, political, nature of a local authority led to unavoidable tensions:

“Sometimes politics and research meet in a way that’s positive and constructive, and sometimes it collides, and sometimes research and objective factual information is inevitably used politically or influenced by politics.” (Interviewee 2).

Pressures arising from the four yearly election cycle were acknowledged. As election time draws closer, Elected Members may begin to look to research for insights into, or solutions for, complex problems, such as health inequalities, but the time required to complete the research process and a need for prompt answers is incompatible. The political landscape may have moved on before research can provide answers, or political priorities changed. Many respondents highlighted the challenge in balancing the need to undertake robust research and the need to complete it quickly, with a tension between ‘academic rigour and the political need to get things done’ (Focus Group 2 Participant 10).

The constraints of the four yearly election cycle also meant that where research and evidence was used to inform decision making, it may be focused on popular, short-term solutions and ‘immediate response’ (Interviewee 4) rather than engaging with the root causes of health inequalities and a longer-term view. The political leaders within the local authority were also felt to be more reluctant to deal with complex problems, such as health inequalities, as they may be viewed as ‘a signal that something isn’t working’ (Focus Group 2 Participant 4) rather than as an opportunity for identifying potential solutions.

Respondents felt this could lead to decisions being made because they would be popular with voters, but that these decisions were made quickly and without establishing what the most appropriate course of action may be:

“There’s a lot going on that you have to do, bang, bang, bang. It’s a bit like that political, you know, this is what we have to do, and we have to do it now. But when do we actually have time to step back and ascertain whether we’ve done the right thing and what have we learnt from it?” (Focus Group 2 Participant 5).

Respondents also described how it was politically expedient to be seen to be operationally focused and pragmatic with a strong focus on day to day delivery of services. Research could, therefore, be something which was a distraction from ‘business as usual’ and be a less attractive option for the use of resources:

“But historically I think there’s a view that research is not doing. So, we’ve become a council that is overly focused on action rather than consideration and careful development of those actions. So, across the organisation I would say it’s kind of frowned upon as being a little bit academic and a little bit of non-delivery.” (Interviewee 6).

The expectation for the local authority to be seen to be focused on delivery also led to constraints on those who had taken on formal training or qualifications, such as an MSc or PhD. On their return to the workforce they are fully committed back into delivery and had little opportunity to use their newly acquired skills:

“I think there’s a lot of people within the [Named] department who are doing their Masters or they have done their Masters, but then it’s incorporating that into the everyday job. And I think sometimes you just revert back to the day job rather than what you’ve actually learnt through doing that programme.” (Focus Group 3 Participant 8).

Barriers arising in the wider political landscape were also identified by respondents. The impacts of austerity and the financial restrictions within which a local authority must operate were widely acknowledged. Time and resource for developing or using research skills and capabilities were limited:

“But again, it’s about how you actually make that happen in terms of resourcing because as the workforce has shrunk, we have less flexibility to enable that to happen without then having to backfill posts.” (Interviewee 7).

Respondents also suggested that the policy and practice of the wider research system was felt to be set up to support academic and NHS organisations conduct research, rather than local authorities:

“So, I’m caveating I suppose that I think academic researchers go through [Professional Network] nationally to then reach individual local authorities. What we don’t do, and there isn’t a system for, is us saying individually or collectively as local authorities here’s an area that we think would benefit from some research and some research expertise, could we collectively put that out to see whether we might find an appropriate research partner to work with us on this? So, it’s a one-way system.” (Interviewee 3).

This potential lack of dialogue could then leave those within the local authority feeling that researchers collected data from the organisation or community and then ‘disappear with it for a couple for years’ (Focus Group 3 Participant 9) without useful outputs coming back into the organisation.

Furthermore, the language used by academics and researchers was not always helpful, or useful, and the perceived ‘elitist world’ (Focus Group 3 Participant 6) of academic research was not considered accessible to the delivery focused local authority.

#### Lack of organisational strategy

Local authorities, as with any organisation, have flux in terms of leadership and strategic direction. Variability in the leadership around research-led decision making presented a number of challenges to tackling health inequalities. Respondents explained that where individual Officers within a service had a personal or professional background or interest in the use of research, then a research-led response to health inequalities may develop. But the use of research was not yet an overarching strategic vision of the organisation.

This ‘patchy and sporadic’ (Interviewee 3) approach to research was problematic. Even where there was a growing interest in the use of research-led decision making amongst practitioners, senior management may not share this position. As senior managers control the service budgets and resources this could then preclude any further action being taken:

“It’s also then about getting buy-in from the highest level, because what’s the point in even trying to look at solutions for a problem if you don’t have buy-in from senior management?” (Focus Group 2 Participant 1).

The lack of a co-ordinated, organisation wide approach to research-led decision making was seen to lead to a culture of research as ‘somebody else’s responsibility’ (Focus Group 1 Participant 2), with services within the local authority providing policy and intelligence functions seen as responsible for providing relevant updates and insights, rather than research-led decision-making being embedded within the organisation.

### Facilitators

Respondents identified several opportunities for research led decision making within the local authority.

#### Recognition of the value of evidence

Respondents described the growing support for research already present within the organisation, and the recognition that the tighter financial constraints required more careful targeting of limited resources for the greatest returns. Research was seen as:

“Spending a little more up front to make sure your finances are focused in the right area.” (Interviewee 6).

In addition, there was a willingness across senior leadership to engage with the culture shift required to take on board the insights available from research, with a growing interest in ‘a bit more thinking about how we could deliver it in practice’ (Interviewee 1).

External research findings were felt to bring the additional advantage of being both instructive for changes to policy and practice while remaining uninfluenced by the possible biases present within the local authority:

“The advantage would be purely that independence, because I know very much, I’m sure, I’m definitely guilty of it, and I’m probably sure other people are, quite often we maybe have a solution in mind before we even start. So, we’re trying to do research that will fit our solution. So, you’ve got that inbuilt bias in the research that you’re doing, so how you ask the questions, who you ask them to, what the content is, you’re almost trying to fit the solution that you’ve got in mind; whereas somebody completely external is probably starting more with a blank piece of paper and is just supplying the evidence that leads you then to a potential solution.” (Focus Group 2 Participant 5).

For a local authority, with the requirement for public consultation and feedback, ‘evidence’ inherently incorporates the ‘local voice’ (Focus Group 3 Participant 6). The value of intelligence generated locally was in the immediate geographical or cultural relevance which fed more easily into any decision making process. As such, respondents reflected the value of co-production to inform decision making around health inequalities. Listening to the voices of the community, and understanding that the use of these insights could result in better service provision and a more efficient use of resources, was driving the focus on evidence based decision making higher up the agenda within the organisation:

“I think there’s also something about leaders understanding what the national agenda and national conversation is around that and engaging with people with lived experience and the value that that can bring to an organisation.” (Focus Group 3 Participant 3).

While acknowledging the cultural differences between the local authority and academics, respondents highlighted the opportunity to drive the use of research when addressing health inequalities by tailoring research findings to the needs of both the Elected Members and Officers separately:

“I think it would be to managers, to me, that it will aid decision making. That if you’ve got the right information, it’s much easier to make decisions on policies. And for politicians as well, the way forward it would be, to me, about helping make decisions.” (Focus Group 1 Participant 7).

Overall, respondents were clear that the challenges of using research in a political organisation were not insurmountable and any research into health inequalities that could ‘bring that strategic and operational-ness together’ (Focus Group 2 Participant 5) would be well received.

#### Building existing networks

Though a divide between the culture and practice of academic, NHS, and local authority organisations was described by respondents, it was also clear that this divide was already being bridged and with further work (on both sides) could be mitigated further. The perception of a divide was manifested in a belief that local authority employees simply did not do research. But some respondents suggested that this was not wholly the case:

“And that was a comment that came back from one of my Service Managers was no we haven’t done any academic research as such. And I said that wasn’t the question that I asked...certainly I had to prompt them to sort of say actually you have done a lot of research and you’ve used that research to put options and recommendations to Elected Members to inform their decisions.” (Interviewee 4).

The national move towards greater integration between local authorities and NHS organisations was also described by respondents as a facilitator of the move towards greater use of research:

“So, I think we’re working on it and we’re trying...because there are two very, very different cultures. So, it’s about understanding each other’s worlds and how we can come together and what we could share, what research we can share that’s applicable to both of us.” (Focus Group 1 Participant 2).

Potential to improve research-led decision making was also felt to lie in the networks around the local authority. Membership of professional networks provided exposure to new ways of working and allowed for the dissemination of research findings:

“I would to some extent try and find out that myself by attending some public lectures at places like the [University], who get a lot of guest speakers in from the Office for National Statistics and the like, to talk about some of the cutting edge stuff that they’re doing.” (Focus Group 3 Participant 9).

Respondents also identified how relationships and networks need to be built with the local voluntary and community sector groups, not just with professional or academic networks, for improved decision making. These groups understood the context and lives of the communities the local authority served and could therefore provide greater insight to help target resources more effectively.

#### Championing a research infrastructure

A champion for research at a senior level was felt to be an important actor to facilitate the growing momentum within the local authority for research and evidence-led decision making. A senior leader would be able to identify where challenges remain in addressing health inequalities, and how to develop services, often as a result of their own background, interest, or simply by ‘being curious’ (Interviewee 7).

Senior leaders could potentially make decisions to fund and support more research. In addition they need to manage the tension between the timescales within which the local authority operates with the timescales of a research process which seeks to create new intelligence. Greater understanding and tolerance of any delays could ultimately lead to the organisation being better informed and able to make more effective decisions about action:

“It’s understanding the timescales, and it’s sometimes you may be asked to look at a problem and they’re expecting a solution very, very quickly, whereas for quality research it’s going to take a prolonged period of time. Obviously within local authority we tend to work in four-year cycles really, if that, coming towards elections and things like that. So, it’s understanding that things don’t happen overnight and that if you want quality information, quality data, it’s going to take time to collect before the solutions can even be dreamt up.” (Focus Group 2 Participant 1).

The economic constraints within which the local authority must operate are unlikely to shift, and limitations on the formal, funded, routes to developing research skills within the workforce are likely to remain. However, respondents identified the informal pathways within the organisation, such as mentoring or secondment, that were available. These could be ‘used more effectively as an organisation’ (Interviewee 6), and, while acknowledging the impacts on resourcing, would bring the benefits to the organisation:

“So likewise, again if a member of my team said do you know what I’d love to spend a day a week with an academic institution researching this, as long as we can make it work in terms of, you know, the pressures that we have, work pressures, then I would really support that.” (Interviewee 5).

## Discussion

This paper sought to understand how research evidence could be more effectively used to inform decision-making in a local authority, focusing particularly on what strengths and assets are currently embedded in the organisational make-up and to identify any potential areas for development. Uniquely, the research design was underpinned by an ER model which has high utility in gaining depth of information and recognising contextual and local factors – we argue that such an innovative methodological approach offers a new contribution to understanding the use of research and evidence in local government. While this ER was largely ‘digitally integrated’, there were particular benefits with adopting a model whereby rapport could be developed with individuals within the local authority to foster rich data gathering. This is discussed again later in this section.

Influenced heavily by evidence-based medicine, evidence-based public health is a long-standing principle of great importance in research and practice. This principle has been amplified by the movement of Public Health into local authorities, with the increasing emphasis on ‘economic rationalism’ and the need to justify expenditure, and ensure that funds are deployed to maximum returns [20]. With the political dimension that local authorities hold, economic rationalism and evidence-based decision making is crucial to ensure democratic legitimacy but, to date, little research exploration has focused on this matter. If local authority personnel are to successfully implement change, then they must draw on the evidence base to aid and support decision-making [21] by elected officials. Indeed, this rhetoric was well understood in this study, and the practical challenges were also recognised by participants.

This study showed the significant challenge for local authority practitioners and policy-makers using evidence to good effect. Some of these issues are unsurprising and have been noted elsewhere [7], it is perhaps axiomatic that busy practitioners working in local authority do not have the space or time to engage in research, evidence generation or assessment and this study re-enforced that this situation has not necessarily changed over time. While this is understandable, it can be a fundamental shortcoming for effective evidence-based decision-making. There is also a strong ethical imperative to adopt the principles of evidence-based practice to ensure that health promotion and public health activity does no harm, either directly or indirectly, by wasting limited funds on ineffective or inappropriate interventions, or by raising unrealistic expectations about what might be achieved. Similar to the findings of the study reported in this paper, in a study by Li et al. [20 , p.196], health promotion practitioners stressed the value of evidence for this reason. One participant in their study noted: ‘I do firmly believe that we need some evidence before we launch into things. I think the prospect of doing harm is too great to not have some inkling of where it is going to go’.

The context of public health within a local authority, a political domain, is also interesting for research and evidence utilisation. Lifestyle drift is the inclination for policy that recognises the need to act on upstream social determinants only to drift downstream to focus on individual lifestyle factors [22]. In a culture where lifestyle interventions are significantly easier to evaluate, and are facilitative of political cycles, it is understandable why more entrenched determinants of health, which takes years to address (i.e. poverty), are often ignored [23]. This strikes to the epicentre of the tension between academic rigour and expedient decision-making and was highlighted here as a common issue in local government. Public health is a very evidence-focused arena, and some have suggested that English local authorities are not a natural home for traditional evidence-based practice. Local government systems are political systems with key decisions needing locally elected officials’ approval [6]. This has direct relevance to research leadership in local authority and having individuals who subscribe to research and evidence-based principles at the pinnacle of local authority structures. The research demonstrated that where this was in place, it fostered stronger commitments to research and evidence-based decision-making within teams and services.

It has been suggested that training for practitioners in interpreting research evidence is a necessary competency to aid professional judgements [24]. Both Li et al. [20] and Owusu-Addo et al. [24] have demonstrated that practitioners in health promotion value evidence from researchers that is context-bound, and relates directly to their own practice, rather than evidence which is more abstract or out-of-context. This was shown in this study where decision-makers had a preference for context-specific evidence. Yet, in reality this can be difficult, and extracting useful evidence from various contexts is critical and does require advanced skills and understanding. The research showed a strong appetite for individuals and groups within the local authority to improve their research skills, and moreover suggested viable ways to do that through training and qualifications and strong connections with academic organisations and institutions. The need for research competency and capacity in local authority is something that is commonly known both nationally and also internationally [25]. Owusu-Addo et al. [24] highlighted that training programmes which build and maintain common skill sets and language among local public health practitioners in Ghana was necessary to accomplish evidence-based public health goals.

The literature highlights the benefits and challenges associated with utilising an ER approach to gather data [15]. Our experience was overwhelmingly positive, in terms of accessing rich and detailed data for analysis and interpretation. The ER approach drew on ethnographic principles, including interviews and observations, but was fundamentally premised on being responsive and agile to opportunities that were presented within the local authority. While the ER was ‘digitally’ embedded and not ‘physically’ embedded as a result of the pandemic, this did not pose significant disadvantage. Indeed, as discussed earlier, in some cases it facilitated expedient access to key personnel who may have otherwise not have been made available. There were, however, some limitations with the study: access and rapport building with employees at the local authority was limited through attendance at pre-arranged meetings and the methods of data collection with limited opportunity for informal conversations, such as those that take place in an office environment; the short-time frame set by funders to set up, deliver and report on the research meant the study team and ER had to focus on ensuring that data collection was prioritised with less time to establish the ER into wider teams across the local authority.

The skill-set of the ER was crucial in being able to navigate both the local authority processes and also the academic collaborators making up the study team. Where challenges arose, they were mitigated by strong partnerships between the research team and the local authority staff (especially those acting as research collaborators) as well as the project steering group. This collective partnership between all constituents worked exceptionally well and enabled data gathering on barriers and facilitators to be conducted relatively smoothly. The ER approach offered the opportunity to gather insight from within the organisation that we are confident would not have been uncovered using other approaches to data gathering.

## Conclusions

The study, utilising a unique ER approach, has explored and shed further insight into the decision-making processes and evidence-based decision-making in local government. Public health practice and practitioners are accustomed to the use of evidence-based decision making, yet this study showed how the democratic and organisational structure of local government challenges how effectively evidence is used in practice. Furthermore, increasing demands, limited capacity and resources impact on even the most research engaged practitioners’ ability to do research. The research highlighted the criticality of research leadership to challenge the status quo in the process of policy development and decision making in local government and move it to one that uses evidence-based principles and prioritises the use and development of research undertaken within local government organisations.

The ER model has high utility in gaining depth of information and recognising contextual and local factors which would support research capacity development in local government. Local government, place based collaborations, and academic institutions should explore and develop opportunities for ERs to bridge the organisational divides, in doing so developing trusted relationships, continued staff development and research capacity.

## Data Availability

The datasets generated and/or analysed during the current study are not publicly available to protect the anonymity of the participants within the local authority but are available from the corresponding author on reasonable request.

## Abbreviations

ER: Embedded Researcher
NHS: National Health Service
NICE: National Institute for Clinical Excellence
LGA: Local Government Association
RCD: Research Capacity Development
NIHR: National Institute for Health Research
